# Exploring the black box of quality improvement collaboratives: modelling relations between conditions, applied changes and outcomes

**DOI:** 10.1186/1748-5908-4-74

**Published:** 2009-11-17

**Authors:** Michel LA Dückers, Peter Spreeuwenberg, Cordula Wagner, Peter P Groenewegen

**Affiliations:** 1 NIVEL - Netherlands Institute for Health Services Research, Utrecht, the Netherlands; 2 EMGO Institute for Health and Care Research, Free University Medical Centre, Amsterdam, the Netherlands; 3 Department of Sociology, Department of Human Geography, Utrecht University, Utrecht, the Netherlands

## Abstract

**Introduction:**

Despite the popularity of quality improvement collaboratives (QICs) in different healthcare settings, relatively little is known about the implementation process. The objective of the current study is to learn more about relations between relevant conditions for successful implementation of QICs, applied changes, perceived successes, and actual outcomes.

**Methods:**

Twenty-four Dutch hospitals participated in a dissemination programme based on QICs. A questionnaire was sent to 237 leaders of teams who joined 18 different QICs to measure changes in working methods and activities, overall perceived success, team organisation, and supportive conditions. Actual outcomes were extracted from a database with team performance indicator data. Multi-level analyses were conducted to test a number of hypothesised relations within the cross-classified hierarchical structure in which teams are nested within QICs and hospitals.

**Results:**

Organisational and external change agent support is related positively to the number of changed working methods and activities that, if increased, lead to higher perceived success and indicator outcomes scores. Direct and indirect positive relations between conditions and perceived success could be confirmed. Relations between conditions and actual outcomes are weak. Multi-level analyses reveal significant differences in organisational support between hospitals. The relation between perceived successes and actual outcomes is present at QIC level but not at team level.

**Discussion:**

Several of the expected relations between conditions, applied changes and outcomes, and perceived successes could be verified. However, because QICs vary in topic, approach, complexity, and promised advantages, further research is required: first, to understand why some QIC innovations fit better within the context of the units where they are implemented; second, to assess the influence of perceived success and actual outcomes on the further dissemination of projects over new patient groups.

## Background

In the last decade, many countries have initiated quality improvement collaboratives (QICs) in healthcare settings. QICs bring together 'groups of practitioners from different healthcare organisations to work in a structured way to improve one aspect of the quality of their service. It involves them in a series of meetings to learn about best practices in the area chosen, about quality methods and change ideas, and to share experiences of making changes in their own local setting' [1]. Another important feature of collaboratives is the use of continuous quality improvement methods to realise changes. Continuous quality improvement is a proactive philosophy of quality management featuring multi-disciplinary teamwork, team empowerment, an iterative approach to problem solving, and ongoing measurement [2,3]. QICs are presented as 'arguably the healthcare delivery industry's most important response to quality and safety gaps', representing substantial investments of time, effort, and funding [4]. Nevertheless, the problem is that despite its popularity, the evidence for QIC effectiveness is positive but limited [3-5]. Effects cannot be predicted with great certainty [6]. Therefore researchers urge for more investigation into the different types of QICs and their effectiveness, as well as linking QIC practices explicitly to organisational and change management theory [1,4,7-9]. Or, as stated by Cretin *et al*., it is important to open the 'black box' of QIC implementation [3].

The current study intends to contribute to a better understanding of the processes and outcomes of QIC implementation in the context of a change programme for 24 Dutch hospitals based on 18 QICs. This programme--a multi-level quality collaborative--is aimed at organisational development and the dissemination of healthcare innovations [10]. It is the third pillar of 'Better Faster', a programme embedded in a broader national policy mix involving an increase in managed competition and transparency, a new reimbursement system based on standardised output pricing, and an intensified role for public actors (like the Healthcare Inspectorate), patient representatives, and healthcare insurers in monitoring the quality and safety of care (see Appendix 1) [10-14]. The multi-level quality collaborative is based on the implementation of different breakthrough collaboratives in the areas of patient safety and logistics. The patient safety targets involve pressure ulcers, medication safety, and postoperative wound infections. Logistics teams deal with operating theatre productivity, throughput times, length of in-hospital stay, and access time for outpatient appointments (for details see Table 1).

**Table 1:** Breakthrough collaboratives and external change agents within Better Faster pillar 3

Quality area	Breakthrough project	Programme targets	Planned year-one projects per hospital
Patient logistics	WWW: working without waiting lists	Access time for out-patient appointments	2
	OT: operating theatre	Increasing the productivity of operating theatres by 30%	1
	PRD: process redesign	Decreasing the total duration of diagnostics and treatment by 40 to 90%, reducing length of in-hospital stay by 30%	2
			
Patient safety	MS: medication safety PU: pressure ulcers	Decreasing the number of medication errors by 50% The percentage of pressure ulcers is lower than 5%	2 2
	POWI: postoperative wound infections	Decreasing postoperative wound infections by 50%	1

Programme hospitals participated for two years in Better Faster pillar 3 (Table 1). During the first year, multi-disciplinary teams in each hospital implemented the following projects that were to be disseminated further in the following year and afterwards [34].

Overview of the breakthrough projects: targets and planned number per hospital in two years

As well as having organisational support provided by the hospitals, each collaborative was organised and facilitated by a small team of external change agents: experts and advisors responsible for the general contents of the projects carried out by the teams in the hospitals. While the multi-level quality collaborative was in its preparation phase, the external change agents served as developers. Their task was to translate promising change ideas into a more or less generally applicable improvement concept, meeting the prerequisites for successful adoption (*e.g*., perceived advantage, low complexity, compatibility [15]). They combined a rapid cycle improvement model with a series of recommended topic related interventions plus performance indicators to monitor progress. Improvement concepts and best practices were transferred at several team training meetings. The teams were trained to apply breakthrough methods, requiring the application of plan-do-study-act improvement cycles and the answering of three questions: 'What are we trying to accomplish?' 'How will we know that a change is an improvement?' and 'What change can we make that will result in an improvement?'[41,42] The one- or two-day training meetings took place at central locations in the county. The agendas contained presentations about background information on the project, team instruction sessions and group assignments, and guest speakers with knowledge about the topic or best practice experience as well as plenary discussion. On average, a delegation of four team members visited four QIC meetings [34].

### Study objective

This study aims to answer two questions: to what extent do expected relationships between conditions, applied changes, and outcomes of QIC-implementation exist; and can differences in conditions and outcomes be explained by the fact that the teams belong to different QICs and hospitals?

### Conceptual framework

This study focuses on relations between relevant conditions for successful QIC implementation, on changes in working methods and activities, and on patient-related outcomes. In opposite order, the outcomes involve perceived project successes and actual progress made in the area of patient safety and logistics. Changes in working methods and activities have to do with all the new or intensified efforts taken by the teams on behalf of their project. The literature on the implementation and dissemination of innovations in health service organisations contains many descriptions of success conditions, linked to the tasks and responsibilities of the actors involved in QIC efforts [15,16]. An important assumption behind QICs as an improvement and spread tool [1] is that knowledge about best practice is made available to teams by external change agents. The teams implement this in their own hospital setting. For this reason, three categories of conditions can be recognised: the organisation of the multi-disciplinary teams that join a QIC and transform the knowledge into action (to avoid confusion, in this study team organisation and teamwork have the same meaning); the degree of support these teams receive from their hospital organisation; and the support given by the external consultants/change agents who facilitate the QIC and its meetings [17].

### Team organisation

This affects the teams joining a QIC. Cohen and Bailey defined a team as 'a collection of individuals who are interdependent in their tasks, who share responsibility for outcomes, who see themselves and who are seen by others as an intact social entity embedded in one or more larger social systems (*e.g*., business unit or corporation), and who manage their relationships across organisational boundaries' [18]. There is a general consensus in the literature that a team consists of at least two individuals who have specific roles, perform interdependent tasks, are adaptable, and share a common goal [19]. To increase team effectiveness, it is important to establish timely, open, and accurate communication among team members [20]. The notion that QIC teams are responsible and in charge of project progress [1] is in line with the literature suggesting that operational decision-making during implementation processes should be devolved to teams [21].

### Organisational support

Other imperatives for team success are strong organisational support and integration with organisational key values [22]. Within QICs, organisational support has to do with the leadership, support, and active involvement by top management [21,23,24]. Regular contact is needed between team and hospital leaders, and the innovation must match the goals of the management [24]. Øvretveit *et al*. state that topics should be of strategic importance to the organisation [1]. In addition to the presence of necessary means and instruments [25], many of the internal support tasks are to be executed by the strategic management. Executives have to communicate a vision or key values throughout the organisation [26,27], and must stimulate the organisation's and employees' willingness to change [28]. These tasks fall within the priority setting areas defined by Reeleeder *et al*.: namely, foster vision, create alignment, develop relationships, live values, and establish processes [29].

### External change agent support

The involvement of external change agents, arranging group meetings for teams of different organisations, is a typical QIC feature. In Table 1, the role of the external change agents within the larger programme is described. Their efforts should contribute to the empowerment and motivation of teams to implement new working methods in order to alter a quality aspect of their care delivery. Team training is a success factor for team-based implementation [22], and can be more effective than individual training, especially when the learning is about a complex technology [30]. External change agents should provide teams with an applicable model together with appealing performance expectations [31]. This implies and requires a gap between a desirable and an actual situation, as well as outlining the potential added value of the innovation to the teams [1]. A second prerequisite is that teams joining the QIC need to gain information and skills that are new to them, otherwise an important argument for joining the QIC is void.

### Hypotheses

**Figure 1:**
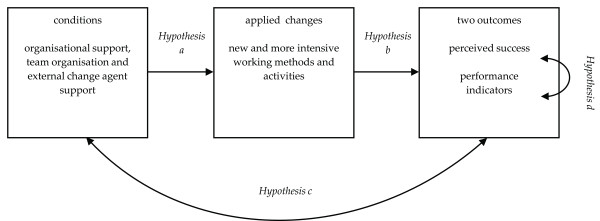
**Study model: hypothesised relations between conditions, applied changes and outcomes**.

In an earlier study, a questionnaire was developed and validated to measure the extent to which these conditions are met [17]. In this article, a model will be tested based on a number of hypotheses that affect the relation between conditions, team-initiated changes due to QIC participation, and two outcome measures (Figure 1).

In the literature, a positive relation is suggested between the presence of these conditions and successful implementation of change [15,16,24]. Successful implementation means that teams manage to adopt new working methods or to alter existing practices. The 18 QICs within the multi-level quality collaborative were aimed at achieving specific targets in the area of patient safety and patient logistics. The implementation of the new working methods and improvement concepts was to be advocated and supported by the external change agents of the QICs. Programme hospitals were expected to provide the necessary internal support. The teams, moreover, were made responsible for the progress of the implementation in their own local hospital setting. Based on the literature and the tasks and responsibilities of actors within the programme in which the QICs are implemented, two hypotheses can be formulated:

Hypothesis A: organisational support, team organisation and external support have a positive effect on the number of applied changes by teams.

Hypothesis B: the number of applied changes has a positive effect on perceived and actual outcomes.

Both hypotheses imply a causal relation. In other instances, it is more difficult to determine the direction of an effect. This applies to hypotheses C and D. Because (A) the number of applied changes is hypothesised to be influenced by the presence of the right conditions and (B) an increase in the number of applied changes has a positive effect on the outcomes, it is logical that (C) the presence of the conditions is expected to be positively related to the outcomes of the implementation:

Hypothesis C: a positive relation exists between conditions and outcomes.

A final assumption has to do with the relation between perceived and actual project outcomes. If an outcome indicator shows that a project's main topic is improved, a project leader is more likely to be positive about the success of the project. Or conversely, if the team leader has a tendency to think more positively about the result, this may have influenced his or her behaviour in such a way that it actually contributed to a higher level of improvement.

Hypothesis D: a positive relation exists between perceived and actual outcomes.

## Methods

### Study population

The total study population consists of 168 teams from 24 hospitals and 18 QICs. Project teams from three hospital groups started, one group after the other, in October 2004, October 2005, and October 2006, with the implementation of the six types of QIC projects as described in Table 1.

### Data sources and variables

Two data sources were accessed to gain information on six variables that were used for the purpose of statistical modelling. The QIC team leaders served as a first data source. In January 2006, 2007, and 2008, the team leaders received a questionnaire at the end of the first year of implementation and were asked to rate the overall success of their project on a scale from zero (min) to ten (max). Other questions reflected relevant conditions for successful implementation. Principal component analysis showed that several of the items measured with the questionnaire (on a seven-point scale) cluster together into three constructs, resembling the categories described in the introduction: organisational support, team organisation, and external change agent support (for information on the items see the notes under Table 2). Scale reliability, internal item consistency, and divergent validity were satisfactory [17]. To measure the number of applied changes, eight activities, relevant for achievement of the project goal, were selected for each QIC from the QIC instruction manuals. Team leaders could mark one out of four options--this is something: we do not do, we have already done, we have intensified/improved since the start of the project, or completely new. For each team, the number of applied changes (intensified/improved or new since the project began) was counted. The applied change rate ranges from zero (no change) to eight (high number of changes).

**Table 2:** The means, medians, inter-quartile ranges (IQR) and ranges of the six variables

**Variable name**:	N	Mean	Median	IQR	Min-Max
External change agent support^1^	168	4.56	4.65	1.46	1.50-6.75
Team organisation^2^	168	5.27	5.40	1.20	1.60-7.00
Organisational support^3^	168	4.60	4.78	1.75	1.40-7.00
Number of applied changes	159	3.73	4.00	2.00	0.00-8.00
Perceived success (overall judgement project leader)	137	6.69	7.00	2.00	1.00-9.00
Actual success (performance indicator)	103	2.28	3.00	2.00	1.00-3.00

^1 ^Items: at collaborative meetings I always gain valuable insights, and external change agents a) provide sufficient support and instruments; b) raise high expectations about performance and improvement potential; c) make clear from the beginning what the goal of the project is and the best way to achieve it; Cronbach's alpha: 0.77.

^2 ^Items: good communication and coordination, clear division of tasks, everyone is doing what he or she should do, team is responsible and in charge of implementation; Cronbach's alpha: 0.84.

^3 ^Items: project is important to strategic management, strategic management supports project actively, hospital gives support needed in the department(s) to make the project a success, board does everything in its power to increase the willingness to change and pays attention to the activities of the project team; Cronbach's alpha: 0.91.

Each QIC served a particular purpose. The external change agents translated project targets into measurable indicators, and teams had to deliver monitoring data to a central database. In this study, these monitoring data were used to model the actual success of the teams. An agreement was made with the organisation funding the programme (as well as the independent evaluation, of which the current study is a part) that the data collection burden for participating hospital staff was to be minimised. Therefore, the central database was the sole source for team performance indicators. Spreadsheet files with team monitoring data were provided three times by the change agency approximately six months after the end of the first implementation year (April to June 2006, 2007, and 2008). These data were used in the analyses that are described later. Project indicators were: prevalence of pressure ulcers (pressure ulcers), prevalence of wound infections (postoperative wound infections), access time for outpatient appointments in days (waiting lists), throughput time for diagnostics and treatment in days (process redesign), and percentage of allocated time actually used (operating theatre productivity). Three types of medication-safety projects had their own indicators: percentage of unnecessary blood transfusions, intravenous antibiotics, or patients with a pain score above four. Medication-safety scores were calculated using the first and last 20 patients treated. Pressure ulcers, operating theatre productivity, and waiting-list project results were based on the change between the scores of the first and last two months. In the case of process redesign and postoperative wound infections, the project period was compared to an identical period in the past.

The change percentages in this study were converted into a three-point scale: (1) at least 10% worse than before, (2) neutral, and (3) improved by at least 10%. Compared to goals such as 30%, 40 to 90% and 50% improvement (Table 1), 10% improvement seems modest. However, several evaluations revealed that even 10% is unrealistic for some teams, making a higher threshold too strict [32,33]. A lower threshold is not an option either, because then the improvement is no longer substantial. It is known from research that an average improvement rate of 10% is common [34], particularly if the improvement strategy--*e.g*., breakthrough--is based on feedback [35].

### Analyses

**Figure 2:**
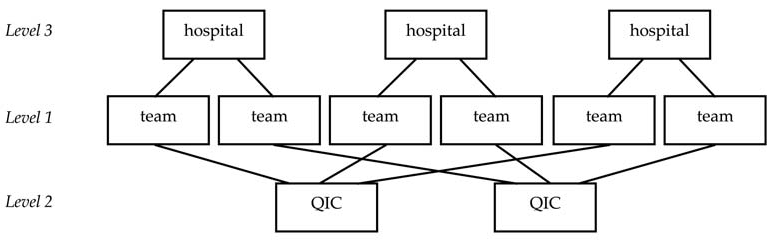
**Cross-classified data structure: project teams nested in QICs and hospitals**.

Multi-level regression analyses were conducted to answer the research questions. The main argument behind multi-level modelling is that social processes often take place within a layered structure. The assumption that data structures are purely hierarchical, however, is often an over-simplification. Entities, such as people or teams, may belong to more than one grouping, and each grouping can be a source of variation. Each team in the current study belongs to one of the 18 QICs and to one of the 24 programme hospitals. For that reason, a cross-classified multi-level model is the most accurate model to study the hypothesised relations between conditions, applied changes and outcomes (Figure 2).

The variance can be separated into three parts: one due to differences between teams (level one), one due to differences between QICs (level two), and one due to differences between hospitals (level three). In the model, the hypotheses were tested in a three-level cross-classified structure as depicted in Figure 2. Intercept variances of all variables were estimated at all three levels. Correlations between the variables were estimated at level one to begin with (given the relatively limited sample size), and at higher levels if the variables belonging to the relations in Figure 1 differed between QICs or hospitals. Five fixed effects were included in the model to test the relation between conditions and applied changes (hypothesis A) and between applied changes and outcomes (hypothesis B).

All analyses were performed using MLwiN software version 2.02. Estimation method was iterated generalised least squares (IGLS) [36].

## Results

A total of 168 team leaders, belonging to 23 hospitals (one hospital refused to participate) and 18 QICs, filled out the questionnaire (71% response rate). Table 2 contains the means, medians, inter-quartile ranges and ranges of the three conditions, the number of applied changes, perceived success, and actual outcome. The number of changed activities was known of 95% of the responding teams (n = 159), overall grades (perceived success) are available with regard to 82% of the teams (n = 137), and 61% of the teams were capable and willing to deliver enough monitoring data to calculate a before and after measurement (actual outcome) (n = 103). Indicator data were available of 94% of the operating theatre productivity teams, 82% of the pressure ulcer teams, 78% of the waiting list teams, 50% of the wound infection teams, 41% of the medication safety teams, and 36% of the process redesign teams.

### Team activities and actual outcomes per project type

The information presented in Table 3 serves as background material. The table shows the number of teams who changed their activities after the project had begun and the average number of applied changes per project type. Pressure ulcer teams mainly applied regular change of patient position (68%) and performed a risk assessment (64%). Medication safety interventions predominantly reflect the three sub-topics the teams dealt with: postoperative pain, blood transfusions, and intravenous antibiotics (29 to 38%). Operating theatre teams focused on starting on time (61%). Wound infection teams reduced the number of door movements and the number of individuals in the operating theatre (89%). They also paid attention to a protocol for optimal administering of antibiotic prophylaxis (61%). Process redesign teams reduced the number of planning moments, reserved slots for specific diagnosis (61%), and clarified decision lines and division of responsibilities (58%). Waiting list teams blocked agendas for six to eight weeks (72%) and anticipated fluctuations (64%). The average number of applied changes per project type ranged from 2.06 (medication safety) to 4.4 (working without waiting lists).

**Table 3:** Activities per breakthrough project: changes implemented during the project (N = 159)

Intensified or new activities to...	More actively or new since beginning of project No. of teams (%)
**Reduce pressure ulcers (28 teams)**	
1. regularly changing patient's position	19 (68%)
2. risk assessment for each patient	18 (64%)
3. patient information brochure on pressure ulcers	16 (57%)
4. compliance to a pressure ulcers protocol	13 (46%)
5. updating the pressure ulcers protocol	12 (43%)
6. occupational and physiotherapy	9 (32%)
7. sufficient anti-pressure ulcers mattresses	6 (21%)
8. specialised pressure ulcer nurse	4 (14%)
Average number of changes (out of eight) applied by pressure ulcer teams	**3.5**

**Improve medication safety (34 teams)**	
1. clinical lesson in pain reduction	13 (38%)
2. spreading a simple card with 'switch' guidelines	12 (35%)
3. reducing postoperative pain; pain score on linear scale <4	11 (32%)
4. reduce degree of unnecessary intravenous antibiotics	10 (29%)
5. compliance to a medication prescription and administering protocol	8 (24%)
6. apply guideline to reduce unnecessary blood transfusion	6 (18%)
7. fixed medication times	4 (12%)
8. double check of all medication	2 (6%)
Average number of changes (out of eight) applied by medication safety teams	**2.0**

**Optimise operating theatre productivity (18 teams)**	
1. starting on time	11 (61%)
2. emergency procedures: re-definition of 'emergency'	8 (44%)
2. reallocate extra operating time based on the degree of utilisation	8 (44%)
4. tracking and solving disturbances in the operating theatre programme	7 (39%)
5. planning based on average surgery time	6 (33%)
5. reduce time between operations	6 (33%)
7. maintaining capacity for emergency available in the programme	5 (28%)
8. staff planning based on differences in surgery time of individual clinicians, differences in anaesthesiologists and assistants, and the experience of the team	2 (11%)
Average number of changes (out of eight) applied by operation theatre teams	**2.9**

**Reduce postoperative wound infections (18 teams)**	
1. limiting the number of persons in the operating theatre	16 (89%)
1. reducing number of door movements	16 (89%)
3. protocol for optimal administering of antibiotic prophylaxis	11 (61%)
4. participation in national wound infections surveillance network	8 (44%)
5. minimise refreshment of bandages	5 (28%)
6. staff reports (skin) infections and diarrhoea	5 (28%)
7. separate working tablet is used for each patient (bandages, instruments, gloves, deposit bags, etc; afterwards cleansing with alcohol)	4 (22%)
8. during wound care no beds are made, nor is the ward cleaned	2 (11%)
Average number of changes (out of eight) applied by wound infections teams	**3.6**

**Reduce throughput times (33 teams)**	
1. reserving slots for specific diagnosis	20 (61%)
1. reducing planning moments	20 (61%)
3. clear decision lines and division of responsibilities	19 (58%)
4. rational planning of demand on expected question	18 (55%)
5. introduction of one-stop shop	16 (48%)
6. admission on day of operation	12 (36%)
6. more flexible staff utilisation	12 (36%)
8. protocol for treatment groups (*e.g*., physiotherapy or informing patients)	11 (33%)
Average number of changes (out of eight) applied by process redesign teams	**3.9**

**Reduce waiting list (36 teams)**	
1. block agendas six or eight weeks in advance; cancellation only in case of emergency	26 (72%)
2. anticipate on fluctuations	23 (64%)
3. minimise types of consults	21 (58%)
3. plan patient consults not routinely but in the event of complaints	21 (58%)
5. perform diagnostics in fewer consults	20 (56%)
6. minimise vacations in busy periods	17 (47%)
7. increase the interval for consultations for chronic disorders	17 (47%)
8. plan realistically on the basis on actual consult length	16 (44%)
Average number of changes (out of eight) applied by waiting list teams	**4.4**

As well as the average changes in activities, the percentage of teams (with data available) experiencing an improvement in the performance indicator by at least 10% also differs between the six project types. This criterion is met by 70% of the pressure ulcer teams (reduction of pressure ulcers), 100% of the medication safety teams, 12% of the operating theatre teams (use of allocated time), 56% of the wound infections teams, 83% of the process redesign teams (throughput times for diagnostics and treatment), and 46% of the waiting list teams (access time).

### Statistical modelling

To learn more about the process and outcomes of QIC implementation, the four hypotheses were tested using multi-level analyses. In Table 4, the estimated correlations, fixed effects, random effects, and the percentage of variance at each level are shown.

**Table 4:** Multi-level model: predicted relations between conditions and outcomes (correlations), associations between applied changes and the conditions and outcomes (fixed effects) and variance components at three levels (random effects)

	Organisational support	Team organisation	External support	Perceived success	Performance indicator
**Correlations**					
Organisational support	-				
Team organisation	0.37^c^	-			
External support	0.25^b^	0.21^a^	-		
Perceived support	0.30^b^	0.29^b^	0.08	-	
Performance indicator	-0.19	0.14	-0.05	-0.08	-
					
**Fixed effects**	Estimate (SE)	Estimate (SE)	Estimate (SE)	Estimate (SE)	Estimate (SE)
(Intercept)	-0.45 (0.16)^b^	-0.11 (0.18)	-0.69 (0.17)^c^	5.57 (0.31)^c^	2.03 (0.21)^c^
Applied changes	0.12 (0.04)^c^	0.04 (0.04)	0.19 (0.04)^c^	0.31 (0.07)^c^	0.09 (0.05)^a^
					
**Random effects**					
Intercept variance at:	Estimate (SE)	Estimate (SE)	Estimate (SE)	Estimate (SE)	Estimate (SE)
- level one (team)	0.69 (0.08)^c^	0.86 (0.11)^c^	0.75 (0.08)^c^	2.01 (0.29)^c^	0.62 (0.11)^c^
- level two (QIC)	0.00 (0.00)	0.07 (0.06)	0.03 (0.04)	0.42 (0.23)^b^	0.15 (0.09)^b^
- level three (hospital)	0.15 (0.07)^a^	0.06 (0.05)	0.08 (0.06)	0.04 (0.12)	0.00 (0.05)
					
Percentage of variance at:					
- level one (team)	82%	87%	87%	81%	81%
- level two (QIC)	0%	7%	3%	17%	19%
- level three (hospital)	18%	6%	9%	2%	0%

^a ^p < 0.05; ^b ^p < 0.01; ^c ^p < 0.001

Note: teams are nested in QICs and hospitals (Figure 2)

Hypothesis A concerns the relation between the three conditions and the number of changes teams applied. The association between organisational support and external change agent support and the number of applied changes is confirmed to be significant (p < 0.001). An increase in organisational support or external change agent support is accompanied by an increase in the number of applied changes. The relation between team organisation and the number of applied changes is insignificant. The multi-level model reveals that organisational support differs significantly between hospitals: 18% of the variance is situated at hospital level. Hypothesis B concerns the effect of applied changes on project outcomes. An increase in the number of applied changes is verified to have a positive effect on perceived success (p < 0.001) and indicator outcomes (p < 0.05). Hypothesis C involves the direct relation between conditions and outcomes. In the case of organisational support and perceived success, and team organisation and perceived success, a positive correlation was found of 0.29 (p < 0.001) and 0.30 (p < 0.001), respectively. The relation between external change agent support and perceived success is not significant (p > 0.05), similar to the relation between the three conditions and actual outcome (p > 0.05). In addition to these test results, a two-tailed Sobel Test was conducted to determine whether the relation between the support conditions and both outcomes is mediated by the number of applied changes [37]. Partial mediation effects were confirmed in the case of organisational support and perceived success (test statistic: 2.77; p < 0.01) and external change agent support and perceived success (test statistic: 3.45; p < 0.001). The mediation of the relationship between conditions and actual outcome is less significant (p < 0.10). At team level, hypothesis D, the existence of a positive relation between perceived success and actual outcome could not be confirmed (p > 0.05). Perceived successes and actual outcomes differ significantly between QICs (p < 0.05). By means of an iterative process, the possibility was explored that the expected hypothesised relation exists at QIC level. After an estimation of the level-two correlation between both variables, the relation could be confirmed: there is a maximal correlation at QIC level (Pearson's r = 1.00; p < 0.05). At this higher group level, perceived successes say more about actual outcomes than at the level of individual teams.

## Discussion

In this article, a model was tested to gain a better understanding of the QIC black box. The study objective was to answer two questions.

### Question 1: Do expected relationships exist between conditions, applied changes, and outcomes?

The analysis resulted in several findings, contributing to a better understanding of the implementation process that took place in the context of the multi-level quality collaborative.

First, when a team leader is more positive about organisational and external change agent support, this has a positive effect on the number of intensified or new working methods applied by the team. Second, a higher number of applied changes has a positive influence on the degree of perceived success and actual outcomes. Third, positive relations between perceived success and organisational support and team organisation could be confirmed. The direct connection between actual outcomes and the three conditions is insignificant. Moreover, the relation between perceived success and organisational support and external change support is partly mediated by the number of applied changes. With regard to the degree of actual success, a similar mediation effect could be verified with 90% certainty.

Finally, the association between actual outcome and perceived success is insignificant at team level but strong at QIC level. The high correlation between perceived and actual success at QIC level indicates that teams who joined a QIC, in which the perceived success ratings of team leaders are high, have also relatively high performance indicator scores.

### Question 2: Are differences in conditions and outcomes due to nesting in hospitals or to QICs?

The multi-level model adds an important dimension that would have been overlooked in a single-level approach. Judgements on external change agent support and team organisation and actual outcomes do not seem to differ between hospitals, but organisational support does. Not one of the conditions differs at QIC level. In the case of external change support, this is particularly interesting because this condition represents the core of the QIC. Apparently, there are no differences in external change agent support between QICs, while at the same time QICs do differ in the level of perceived and actual success. Nevertheless, the finding that an increase in external change agent support is accompanied by an increase in the number of applied changes confirms the relevance of external change agents within QICs as a mechanism for best practice transfer.

### Implications

It was mentioned in the introduction that the evidence on QIC effectiveness is mixed but positive. Mittman explained how subjective ratings provided by collaborative participants and leaders are subject to unintentional and unrecognised biases generated by common human decision and judgment heuristics. In that respect, he exemplified how a combination of expectation biases and belief perseverance produces systematic overweighting of evidence and observations. *A priori *expectations and beliefs are confirmed, while evidence that does not support the effectiveness of the QIC method is underweighted or discounted [4]. This study confirms the risk addressed by Mittman. The overall judgement of an individual team leader is confirmed to say little about actual indicator outcomes and vice versa. This is not necessarily a bad thing--at least as long as the evaluation goal is not about assessing cost effectiveness or public accountability of the means invested in QIC programmes. Still, parties involved in implementing QIC projects should be cautious when it comes to rating and explaining the merits of their work, especially when monitoring data are not yet available. This also applies to QIC researchers who use perceived successes as proxy variables for actual performance. The overall success judgement apparently represents something other than monitored progress towards project goals. Like the actual outcomes, it depends on the number of applied changes. It is also likely that team leaders base their success judgement on other accomplishments: for instance, they notice how patients benefited from the project or how the team managed to change old routines and implemented new interventions that are expected to pay off in the long run.

The study confirms the association between organisational and external change agent support and the number of changes realised by QIC teams. Hospital managers, project teams, external change agents, and public stakeholders may benefit from the survey instrument, because it potentially provides tangible information, applicable for real-time adjustments or intake procedures.

Researchers are in a situation where relevant questions remain unanswered. Generally, the advice to adopt hierarchical models in future research should be taken as seriously, as are recommendations for more experimental [7], narrative [15], or action-based research studies [38]. Further research is needed to test the effectiveness of QICs as spread strategy [1] and to assess how external change agent support influences team organisation, how team learning within a QIC takes place, and how QICs contribute to organisational learning. In addition to the black box of QIC implementation, there is another black box that needs to be opened: that of sustainability. In the extensive 'diffusion of innovation' review, Greenhalgh *et al*. found many studies addressing adoption, implementation, and diffusion, but only a limited number of studies dealing with sustainability [15].

### Strengths and weaknesses

The multi-level approach is one of the strengths of this study. Other strengths are that the conditions were measured using a validated and reliable instrument, and perceptions were linked to outcome data. The dependence on data provided by the teams is a limitation. Despite the high response rate, the use of self-reported perceptions always involves a risk of overestimation or social desirability. Outcome indicators could be linked to questionnaire data in 61% of all teams in the study sample. It is very likely that the positive results are overrepresented, particularly because the absence of monitoring data may very well be caused by the fact that teams were incapable of implementing the project (and the required measurements) as planned. In that sense, actual outcomes presented in this article do not entirely represent the overall level of success of the programme.

While the vast majority of the projects had a planned length of one year, operation theatre, process redesign, and postoperative wound infections were in fact two-year projects. Because the team questionnaires were administered at a fixed moment by the end of the first year, second-year data on conditions, perceived success, and applied changes are unfortunately unavailable. Hence, for practical reasons, the analyses described in this article are based entirely on first-year data. A potential limitation is that the success level of two-year projects was determined without the project being finished. At first glance, it is reasonable to assume that the improvement rate of those projects is likely to be more positive after two years. A recent evaluation, however, illustrates that the level of improvement has remained the same [39]. An additional analysis would yield similar results.

Finally, the number of applied changes was modelled without taking into account the influence of individual and key interventions or specific combinations. In reality, some interventions are more time-consuming and complex than others, and some may not even be suited for application within a collaborative [39].

## Summary

By examining 18 QICs, part of a quality improvement programme for hospitals, several expected relationships could be verified. Organisational and external change agent support had a positive influence on the number of changes applied by QIC teams during the implementation. The number of applied changes had a positive effect on perceived success as well as on actual outcomes. By taking into account the fact that teams are nested in hospitals and in QICs, it became clear that some hospitals are better than others in providing organisational support. Project outcomes differ between QICs. One should be cautious when accepting perceived successes as a proxy for the actual success of individual teams.

## Competing interests

The authors declare that they have no competing interests.

## Authors' contributions

MLAD was responsible for designing the study, acquiring, analyzing and interpreting the data, and drafting the manuscript. PS assisted with the analyses and interpretation of the data. As research manager of the independent evaluation study of the hospital improvement programme, CW was responsible for designing the study. CW and PPG assisted in interpreting the results and revising the manuscript for intellectual content. All authors have read and approved the final manuscript.

## Appendix 1 - Description of the three pillars of Better Faster

### Pillar 1

The purpose of the first pillar was to create awareness and provide room for new paradigms by having authoritative experts from other fields of service delivery and industry communicate appealing approaches and ideas about how to deal with issues of safety, logistics, and accountability in healthcare. Focus was added to national and local discussions on necessary changes[10].

### Pillar 2

Transparency is thought to guide purchasing decisions and improvement efforts. The second pillar is considered an important step in generating comparative data on healthcare quality. A national set of standardised quality indicators for hospital care has been developed and maintained by the Healthcare Inspectorate [11].

### Pillar 3

A national programme to stimulate transparency, efficiency, and quality of care was implemented in three groups of eight hospitals between 2004 and 2008, covering approximately a quarter of all hospitals in the Netherlands. This multi-level quality collaborative combined interventions at the bottom and the top of member organisations.

At bottom level, physicians, nursing staff, and managers were encouraged to participate in quality improvement collaboratives to continuously improve the quality of their work by trying out interventions using a breakthrough model while being supported by their institution and by external change agents (Table 1).

At top level, hospital executives participated in a special collaborative leadership programme (leadership and organisational development). An internal programme organisation was established to monitor the progress of the various programmes. The strategic management was expected to encourage active staff participation [40] and to support the implementation and spread of the new working methods and results. Feedback loops were established at unit and process level, part of the learning cycles during the implementation of the breakthrough model. In addition, the leadership programme strived explicitly for realisation of feedback loops at institutional level to promote the congruence between strategic hospital goals and the performance at unit level [10].
